# The Association of Serum AIM2 Level with the Prediction and Short-Term Prognosis of Coronary Artery Disease

**DOI:** 10.1155/2022/6774416

**Published:** 2022-05-14

**Authors:** Wenkang Zhang, Gaoliang Yan, Chunyang Xu, Chengchun Tang

**Affiliations:** ^1^School of Medicine, Southeast University, Nanjing, Jiangsu 210009, China; ^2^Department of Cardiology, Zhongda Hospital, School of Medicine, Southeast University, Nanjing, Jiangsu 210009, China

## Abstract

**Objective:**

Coronary artery disease (CAD), one of the commonest cardiovascular diseases, has high morbidity and mortality. Absent in melanoma 2 (AIM2) is involved in atherosclerosis, and no clinical trials have explored the association between AIM2 and CAD. Therefore, this study was aimed at evaluating the predictive and short-term prognostic value of AIM2 for CAD.

**Methods:**

279 patients who underwent coronary angiography were enrolled in this study. The AIM2 level was detected from the serum of collected artery blood samples. The association of serum AIM2 level with the prediction and short-term prognosis of CAD was further assessed.

**Results:**

The serum AIM2 level of the CAD group was significantly higher than the control group (5.5 ± 2.1 vs. 3.7 ± 1.7; *p* < 0.001). AIM2 was demonstrated to be the risk factor of CAD [odds ratio, 1.589; 95% confidence interval (CI), 1.346-1.876; *p* < 0.001]. The area under the receiver operating characteristic (ROC) curve of 0.738 showed the diagnostic value of AIM2 in CAD. Additionally, AIM2 was an independent predictor of major adverse cardiovascular events (hazard ratio, 1.453; 95% CI, 1.086-1.945; *p* = 0.012), and CAD patients with high AIM2 levels (>4.9 ng/mL) had a markedly lower survival rate (log-rank *p* = 0.040).

**Conclusions:**

The serum AIM2 level > 4.9 ng/mL can predict CAD to a certain extent. AIM2 might be an independent predictor of its short-term poor prognosis.

## 1. Introduction

Coronary artery disease (CAD), one of the commonest cardiovascular diseases, has high morbidity and mortality, which threatens people's life and health [1]. Due to the complex pathogenesis of CAD, atherosclerosis is the basis [2].

Absent in melanoma 2 (AIM2), the receptor of cytosolic innate immune, can recognize and bind with double-stranded DNA (dsDNA), leading to AIM2, apoptosis-associated speck-like protein (ASC) and caspase-1 assembling to form AIM2 inflammasome [3]. After AIM2 inflammasome activation, N-terminal domain of gasdermin D (GSDMD-NT) released from GSDMD via caspase-1 cleavage eventually results in cell pyroptosis and proinflammatory factor interleukin-1*β* (IL-1*β*) and interleukin-18 (IL-18) release, which is involved in the initiation and progression of atherosclerosis [4, 5].

Recent studies have confirmed that AIM2 is related to atherosclerosis, myocardial infarction, heart failure, and abdominal aortic aneurysm [5]. Hence, we believe that AIM2 might play a pivotal role in cardiovascular diseases. However, no clinical trials have further explored the association between AIM2 and CAD. Therefore, in this study, we first detected the serum level of AIM2 in CAD patients and further analyzed the association of serum AIM2 level with the prediction and short-term prognosis of CAD.

## 2. Materials and Methods

### 2.1. Study Population

279 patients who underwent coronary angiography (CAG) in Zhongda Hospital, Southeast University, between July 2020 and July 2021, aging from 18 to 85, were enrolled in this study. Followed by the diagnostic criteria of the American Heart Association, 183 patients were diagnosed with CAD and separated into the CAD group. Non-CAD was confirmed in the control group of 96 individuals. The following were exclusion criteria: myocarditis, cardiomyopathy, valvulopathy, severe cardiac, hepatic and renal dysfunction, autoimmune diseases, hematologic disorders, peripheral vascular diseases, infectious diseases, malignancies, allergy to iodine and iodinated contrasted medium, and loss to follow-up. The current study was approved by the institutional ethics committee for clinical research of our hospital (No. 2020ZDSYLL207-Y01). All selected participants signed a written informed consent.

### 2.2. Baseline Characteristics Collection and AIM2 Assay

Baseline characteristics, including demographic data, past histories, echocardiography, laboratory parameters, and medications, were recorded by experienced clinicians. Venous blood samples for testing laboratory parameters were collected after overnight fasting and assayed by automatic biochemistry analyzers in the clinical laboratory. Serum was obtained from centrifugation of collected artery blood samples (5 mL) before CAG and maintained at -80°C refrigerator. The serum AIM2 level was detected by human AIM2 ELISA kits (Meimian Biotechnology, Yancheng, China).

### 2.3. Follow-Up and Endpoints

All patients had a 180-day follow-up for major adverse cardiovascular events (MACEs), including cardiogenic death, target vessel revascularization (TVR), heart failure, nonfatal myocardial infarction, and stroke, and all these data were acquired through returning visits or telephone.

### 2.4. Statistical Analysis

Data were analyzed using SPSS software version 25.0 (SPSS Inc., Chicago, IL, USA). Continuous variables were summarized using mean ± standard deviation or median (IQR) according to normal distribution or not, and categorical variables using frequencies and percentages. Differences in continuous variables were compared by an independent sample *t*-test or Mann–Whitney *U*-test, and categorical variables by chi-square test or Fisher's exact test. Pearson's or Spearman's correlation test was performed to reveal the correlation between AIM2 and CAD risk factors. Univariate and multivariate logistic regression analyses were applied to judge the risk factors of CAD. The receiver operating characteristic (ROC) curve was depicted to calculate the sensitivity, specificity, cut-off value of AIM2 in CAD diagnosis, and the area under the curve (AUC). MACE-free survival was analyzed using the Kaplan-Meier curve. The univariate and multivariate Cox proportional hazards regression analyses were examined to determine whether AIM2 is an independent predictor of MACEs. Two-sided *p* values of <0.05 were considered statistically significant.

## 3. Results

**Figure 1:**
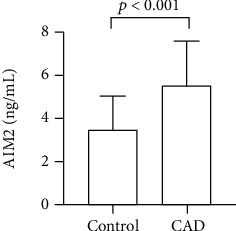
The serum AIM2 levels in the CAD group and the control group.

Baseline study population characteristics are shown in Table 1. Compared with the control group, patients in the CAD group had higher white blood cell (WBC) and cystatin C levels (all *p* < 0.05). In addition, significant differences in several medications, like aspirin, adenosine diphosphate (ADP)-receptor blocker, statin, beta-blocker, and diuretic (all *p* < 0.05), were also observed in the CAD and control group. What is more, the serum AIM2 level of the CAD group was significantly higher than that of the control group (5.5 ± 2.1 vs. 3.7 ± 1.7; *p* < 0.001) (Figure 1).

The association of AIM2 with the CAD risk factors was further analyzed. Correlation analysis found that the serum AIM2 level was positively correlated to smoke (*r* = 0.171, *p* = 0.021), low-density lipoprotein cholesterol (LDL-C) (*r* = 0.256, *p* < 0.001), and serum creatinine (Scr) (*r* = 0.185, *p* = 0.012) (Table 2). Univariate and multivariate logistic regression analyses both showed that AIM2 [odds ratio (OR), 1.615; 95% confidence interval (CI), 1.386-1.881; *p* < 0.001; OR, 1.589; 95% CI, 1.346-1.876; *p* < 0.001, respectively], age (OR, 1.030; 95% CI, 1.006-1.054; *p* = 0.013; OR, 1.041; 95% CI, 1.011-1.072; *p* = 0.007, respectively), and LDL-C (OR, 1.847; 95% CI, 1.297-2.631; *p* = 0.001; OR, 1.607; 95% CI, 1.071-2.412; *p* = 0.022, respectively) were the risk factors of CAD (Table 3).

**Figure 2:**
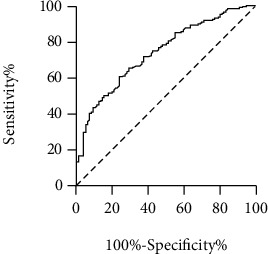
Receiver operating characteristic (ROC) curve of AIM2 on predicting CAD.

On ROC curve analysis (Figure 2), the AUC was 0.738 (95% CI: 0.679-0.797, *p* < 0.001), and the sensitivity and specificity of AIM2 were 60.1% and 76.0%, respectively, when the optimum cut-off was 4.9 ng/mL, demonstrating the diagnostic value of AIM2 in CAD.

**Figure 3:**
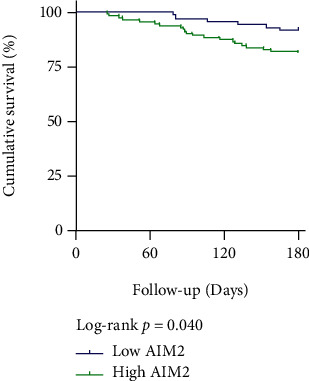
Kaplan-Meier curves for survival analysis of MACE-free survival.

Then, the groups were divided into low (≤4.9 ng/mL, *n* = 73) and high (>4.9 ng/mL, *n* = 110) AIM2 level groups by the cut-off value of AIM2 level to study the effect of AIM2 on clinical outcomes of CAD.  Table 4 shows that, during the 180-day follow-up, MACEs happened in 21 (19.1%) subjects in the high AIM2 level group and 6 (8.2%) subjects in the low AIM2 level group, and the difference was significant (*p* = 0.042). No significant differences were found between the two groups in cardiogenic death, TVR, heart failure, nonfatal myocardial infarction, and stroke. Kaplan-Meier survival analysis exhibited that CAD patients with high AIM2 levels (>4.9 ng/mL) had a markedly lower survival rate (log-rank *p* = 0.040) (Figure 3).

The univariate Cox proportional hazards regression analysis suggested that AIM2 was significantly associated with MACEs [hazard ratio (HR), 1.383; 95% CI, 1.144-1.672; *p* = 0.001]. What is more, after adjusting for age and male in model 1 and age, male, smoke, hypertension, DM, hyperlipidemia, LDL-C, and Gensini score in model 2, AIM2 was still an independent predictor of MACEs in both adjusted models 1 (HR, 1.430; 95% CI, 1.173-1.745; *p* < 0.001) and 2 (HR, 1.453; 95% CI, 1.086-1.945; *p* = 0.012) (Table 5).

## 4. Discussion

Former strong evidence has shown that AIM2 was involved in atherosclerosis, and a high level of AIM2 was expressed in atherosclerotic plaque of the human carotid artery and mice [4, 6–8]. However, as of yet, the clinical significance of the serum AIM2 level in CAD patients has not been identified. Our study first revealed that the serum AIM2 level of CAD patients was higher than that of controls. Of note, AIM2 was positively correlated to LDL-C, Scr, and smoke, the CAD risk factors, and was the risk factor of CAD. These can be explained as follows: (i) atherogenic risk factors, like LDL-C and high glucose, and cytokines involved in atherosclerosis, like tumor necrosis factor-alpha (TNF-*α*) and interferon-*γ* (IFN-*γ*), can upregulate AIM2 expression [8–12]. (ii) AIM2 can enhance coronary artery endothelial cell injury, endothelial-monocyte adherence, monocyte recruitment, and macrophage accumulation in pathological parts [4, 12–14]. (iii) Migration from the vascular media to the intima of vascular smooth muscle cells (VSMCs) can be facilitated by AIM2 [6]. (iv) AIM2 inflammasome activation and atherogenic proinflammatory factor interleukin-1*β* (IL-1*β*) and interleukin-18 (IL-18) release promote inflammatory responses of atherosclerosis [7, 12]. (v) Larger necrotic core and thinner fibrous cap caused by VSMC pyroptosis and inflammation via AIM2 decrease plaque stability [4, 7, 15, 16].

The relationship between the AIM2 level and clinical outcomes of CAD remains unclear. In recent studies, Li et al. [17] found that upregulation of AIM2 aggravated myocardial damage in ischemia/reperfusion (I/R) mice. Devi et al. [18] revealed that the AIM2 inflammasome activation was associated with maladaptive left ventricle (LV) remodeling and dysfunction after myocardial infarction. Wang et al. [10] found that diabetic rats with higher AIM2 levels in heart induced by streptozotocin showed severe left heart insufficiency, including metabolic block, cardiomyocyte death, and myocardial fibrosis. Therefore, AIM2 was to be related to cardiac structure and function, which might affect the prognosis of CAD. The present study found that MACEs were more prone to be existed in patients with high AIM2 levels. More to the point, after the addition of various risk factors, AIM2 was still an independent predictor of MACEs. As above, the AIM2 level had close relationship with short-term cardiovascular events.

The research above may suggest that the new therapy of AIM2 for CAD is full of promise. Paulin et al. [7] found that the application of AIM2-antagonizing synthetic oligonucleotide A151 on hypercholesterolemic mice reduced VSMC death and necrotic core size, promoted lesional collagen deposition, and accelerated fibrous cap thickening, which decreased plaque vulnerability. Li et al. [17] found that using sevoflurane in myocardial I/R mice could reduce infarct size and cardiomyocyte apoptosis through the downregulated AIM2 expression in the myocardium. Although these discoveries have confirmed its feasibility in animal experiments, there have been no safe and practicable human tests until now, and it remains to be seen whether the same results can be obtained in human trials. What is more, pharmacological inhibition of AIM2 might produce side effects responsible for less inflammatory responses mediated by AIM2 inflammasome, which is also worthy of consideration.

Several limitations existed in our study. First, its single-center performance, the small sample size, and the short follow-up time tended to impact the reliability of the results. Second, AIM2 may affect atherosclerosis through inflammation via inflammasome, but some involved inflammatory indicators were not detected. Finally, AIM2 made a difference to the plaque stability, but the relation between AIM2 and plaque vulnerability in CAD patients was not investigated owing to the lack of plaque evaluation via instruments. Therefore, advanced researches with more subjects and longer follow-up times are required to validate these findings further, and additional evaluations of related inflammatory cytokines and plaque vulnerability would make the discoveries much more sense.

## 5. Conclusions

In conclusion, this study demonstrated that the serum AIM2 level > 4.9 ng/mL can predict CAD to a certain extent. Also, AIM2 might be an independent predictor of its short-term poor prognosis.

## Figures and Tables

**Table 1:** Baseline characteristics of the study population.

Variables	Control group (*n* = 96)	CAD group (*n* = 183)	*p* value
Age (years)	63 ± 10	65 ± 11	0.053
Male (*n*, %)	54 (56.3)	118 (64.5)	0.179
BMI (kg/m^2^)	25.2 ± 3.8	25.8 ± 3.6	0.218
SBP (mmHg)	131 ± 16	135 ± 21	0.054
DBP (mmHg)	78 ± 14	79 ± 13	0.507
HR (bpm)	78 (70, 88)	78 (70, 86)	0.368
Smoke (*n*, %)	27 (28.1)	66 (36.1)	0.181
*Past histories*			
Hypertension (*n*, %)	61 (63.5)	135 (73.8)	0.076
DM (*n*, %)	20 (20.8)	57 (31.1)	0.067
Hyperlipidemia (*n*, %)	9 (9.4)	32 (17.5)	0.069
Af (*n*, %)	4 (4.2)	15 (8.2)	0.204
Stroke (*n*, %)	15 (15.6)	41 (22.4)	0.179
*Echocardiography*			
LA (cm)	3.8 (3.5, 4.1)	3.9 (3.6, 4.2)	0.050
LV (cm)	4.6 (4.4, 4.9)	4.7 (4.3, 5.1)	0.440
LVEF (%)	67 (63, 71)	66 (60, 70)	0.076
*Laboratory parameters*			
WBC (×10^9^/L)	5.7 (4.8, 6.6)	6.8 (5.6, 8.2)	<0.001
RBC (×10^12^/L)	4.7 (4.2, 5.0)	4.6 (4.2, 4.9)	0.700
Hb (g/L)	137 (127, 144)	138 (126, 149)	0.510
Plt (×10^9^/L)	221 ± 66	209 ± 56	0.141
Hct (%)	41.8 ± 5.3	41.3 ± 4.8	0.481
TSB (*μ*mol/L)	12.7 (10.5, 16.2)	11.9 (9.5, 15.9)	0.092
ALT (*μ*mol/L)	25 (17, 32)	23 (18, 34)	0.917
AST (*μ*mol/L)	25 (19, 33)	23 (19, 32)	0.585
BUN (mmol/L)	5.9 (4.8, 7.2)	5.9 (4.8, 7.1)	0.933
Scr (*μ*mol/L)	67 (58, 78)	68 (57, 82)	0.490
UA (*μ*mol/L)	319 (287, 377)	331 (266, 406)	0.741
Cystatin C (mg/L)	0.8 (0.7, 1.0)	0.9 (0.8, 1.1)	<0.001
TG (mmol/L)	1.5 (1.0, 2.1)	1.2 (0.9, 1.8)	0.091
TC (mmol/L)	3.6 (3.0, 4.2)	3.7 (3.0, 4.5)	0.261
HDL-C (mmol/L)	1.2 (1.0, 1.3)	1.1 (1.0, 1.3)	0.328
LDL-C (mmol/L)	2.0 (1.6, 2.4)	2.1 (1.6, 2.6)	0.388
Apoa (mg/L)	139 (75, 252)	120 (58, 254)	0.394
eGFR-MDRD (mL/min × 1.73 m^2^)	101.3 (85.5, 110.7)	101.3 (84.0, 118.9)	0.814
*Medications*			
Aspirin (*n*, %)	0 (0.0)	165 (90.2)	<0.001
ADP-receptor blocker (*n*, %)	0 (0.0)	136 (74.3)	<0.001
Statin (*n*, %)	6 (6.3)	178 (97.3)	<0.001
Beta-blocker (*n*, %)	20 (20.8)	134 (73.2)	<0.001
ACEI/ARB (*n*, %)	36 (37.5)	86 (47.0)	0.129
CCB (*n*, %)	31 (32.3)	57 (31.1)	0.845
Diuretic (*n*, %)	13 (13.5)	49 (26.8)	0.012

Data are presented as *n* (%), mean and standard deviation, or median (25th, 75th). CAD: coronary artery disease; BMI: body mass index; SBP: systolic blood pressure; DBP: diastolic blood pressure; HR: heart rate; DM: diabetes mellitus; Af: atrial fibrillation; LA: left atrium; LV: left ventricle; LVEF: left ventricular ejection fraction; WBC: white blood cell; RBC: red blood cell; Hb: hemoglobin; Plt: platelet; Hct: hematocrit; TSB: total serum bilirubin; ALT: alanine aminotransferase; AST: aspartate aminotransferase; BUN: blood urea nitrogen; Scr: serum creatinine; UA: uric acid; TG: triglyceride; TC: total cholesterol; HDL-C: high-density lipoprotein cholesterol; LDL-C: low-density lipoprotein cholesterol; Apoa: apolipoprotein (a); eGFR-MDRD: estimated glomerular filtration rate based on MDRD equation; ADP: adenosine diphosphate; ACEI: angiotensin-converting enzyme inhibitor; ARB: angiotensin receptor blocker; CCB: calcium channel blocker.

**Table 2:** Correlation between AIM2 and CAD risk factors.

Risk factors	*r*	*p* value
Male	0.069	0.351
Age	0.037	0.620
BMI	0.027	0.713
Hypertension	0.075	0.312
DM	0.081	0.273
Smoke	0.171	0.021
TG	0.134	0.071
TC	0.096	0.197
LDL-C	0.256	<0.001
Scr	0.185	0.012

BMI: body mass index; DM: diabetes mellitus; TG: triglyceride; TC: total cholesterol; LDL-C: low-density lipoprotein cholesterol; Scr: serum creatinine.

**Table 3:** Logistic regression analysis results for risk factors of CAD.

Variables	Univariate			Multivariate		
	OR	95% CI	*p* value	OR	95% CI	*p* value
Age	1.030	1.006-1.054	0.013	1.041	1.011-1.072	0.007
Male	1.412	0.853-2.338	0.180	1.205	0.643-2.258	0.562
BMI	1.044	0.975-1.118	0.218	1.034	0.954-1.120	0.419
Hypertension	1.614	0.950-2.742	0.077	1.417	0.765-2.625	0.268
DM	1.719	0.959-3.081	0.069	1.300	0.672-2.516	0.436
Smoke	1.442	0.842-2.468	0.182	1.291	0.653-2.552	0.462
AIM2	1.615	1.386-1.881	<0.001	1.589	1.346-1.876	<0.001
LDL-C	1.847	1.297-2.631	0.001	1.607	1.071-2.412	0.022
Scr	1.012	0.998-1.026	0.087	1.007	0.991-1.023	0.416

OR: odds ratio; CI: confidence interval; BMI: body mass index; DM: diabetes mellitus; AIM2: absent in melanoma 2; LDL-C: low-density lipoprotein cholesterol; Scr: serum creatinine.

**Table 4:** Clinical outcomes between two groups.

Outcomes variables (*n*, %)	Groups by AIM2 cut-off value (ng/mL)		*p* value
	Low (≤4.9, *n* = 73)	High (>4.9, *n* = 110)	
MACEs	6 (8.2)	21 (19.1)	0.042
Cardiogenic death	0 (0.0)	2 (1.8)	0.518
Nonfatal MI	2 (2.7)	5 (4.5)	0.818
TVR	0 (0.0)	2 (1.8)	0.518
HF	3 (4.1)	9 (8.2)	0.433
Nonfatal stroke	1 (1.4)	3 (2.7)	0.921

AIM2: absent in melanoma 2; MACEs: major adverse cardiovascular events; MI: myocardial infarction; TVR: target vessel revascularization; HF: heart failure.

**Table 5:** Cox regression analysis for AIM2 and MACEs.

Models	AIM2	
	HR (95% CI)	*p* value
Unadjusted	1.383 (1.144-1.672)	0.001
Adjusted model 1	1.430 (1.173-1.745)	<0.001
Adjusted model 2	1.453 (1.086-1.945)	0.012

AIM2: absent in melanoma 2; HR: hazard ratio; CI: confidence interval.

## Data Availability

Data related to this paper can be made available from the corresponding author upon reasonable request.
